# In Vitro and In Vivo Gastrointestinal Survival of Non-Encapsulated and Microencapsulated *Salmonella* Bacteriophages: Implications for Bacteriophage Therapy in Poultry

**DOI:** 10.3390/ph14050434

**Published:** 2021-05-06

**Authors:** Laura Lorenzo-Rebenaque, Danish J. Malik, Pablo Catalá-Gregori, Clara Marin, Sandra Sevilla-Navarro

**Affiliations:** 1 Departamento de Producción y Sanidad Animal, Salud Pública Veterinaria y Ciencia y Tecnología de los Alimentos, Instituto de Ciencias Biomédicas, Facultad de Veterinaria, Universidad Cardenal Herrera-CEU, CEU Universities, Calle Tirant lo Blanc, 7, 46115 Alfara del Patriarca, Spain; laura.lorenzorebenaque@uchceu.es (L.L.-R.); p.catala@cecav.org (P.C.-G.); s.sevilla@cecav.org (S.S.-N.); 2 Chemical Engineering Department, Loughborough University, Loughborough LE11 3TU, UK; d.j.Malik@lboro.ac.uk; 3 Centro de Calidad Avícola y Alimentacion Animal de la Comunidad Valenciana (CECAV), 12539 Castellón, Spain

**Keywords:** bacteriophages, *Salmonella*, poultry, Eudragit^®^, encapsulation

## Abstract

The therapeutic use of bacteriophages is recognized as a viable method to control *Salmonella*. Microencapsulation of phages in oral dosage forms may protect phages from inherent challenges of the gastrointestinal tract in chickens. Therefore, the main objective of this study was to assess the survival of *Salmonella* BP FGS011 (non-encapsulated and microencapsulated) through the gastrointestinal tract under in vitro as well as in vivo conditions after oral administration to 1-day-old chicks. To this end, the phage FGS011 was encapsulated in two different pH-responsive formulations with polymers Eudragit^®^ L100, and Eudragit^®^ S100 using the process of spray drying. Phages encapsulated in either of the two formulations were able to survive exposure to the proventriculus-gizzard in vitro conditions whereas free phages did not. Moreover, phages formulated in polymer Eudragit^®^ S100 would be better suited to deliver phage to the caeca in chickens. In the in vivo assay, no statistically significant differences were observed in the phage concentrations across the gastrointestinal tract for either the free phage or the encapsulated phage given to chicks. This suggested that the pH of the proventriculus/gizzard in young chicks is not sufficiently acidic to cause differential phage titre reductions, thereby allowing free phage survival in vivo.

## 1. Introduction

Non-typhoidal *Salmonella* spp. has been recognized as one of the most important zoonotic pathogens worldwide [1,2]. Latest data reported by the World Health Organization estimated 78 million annual cases of foodborne illness worldwide, of which 59,000 resulted in death [1]. By the same token, in the European Union (EU), the latest data published in 2019, revealed more than 90,000 cases of human salmonellosis [3]. *Salmonella* sources in human infection are relatively diverse, however poultry is considered a major reservoir [3,4,5,6]. Due to this situation, since 2008, National *Salmonella* Control Programs (NSCP) in accordance with Regulation (EC) No 2160/2003 have been implemented in the EU [7,8]. These measures have resulted in significant *Salmonella* prevalence reduction in poultry flocks throughout Europe [9]. However, some *Salmonella* serovars related to food-borne outbreaks are still present in some poultry farms, due to their ability to survive and grow in the gastrointestinal tract (GIT) of chickens and/or farm environment [4,10,11,12]. When *Salmonella* comes into contact with the bird and reaches the GIT, its ability to rapidly colonize and multiply results in long-term bacterial excretion in faeces especially in 1-day-old chicks [13,14,15,16]. *Salmonella*, almost colonizes every part of the chicken GIT, nevertheless, the pH of the GIT could affect the bacteria colonization [17]. *Salmonella* encounters the acidic environment of the crop, with a pH of 5, that is maintained and controlled by the bacteria of the genus *Lactobacillus* [17]. Subsequently, *Salmonella* survives the low pH of the proventriculus and gizzard (pH 2.5), and colonize the gut (pH 5.5–8), with highest predilection potential noticed in the ceca [18,19,20]. Thus, innovative and cost-effective techniques are needed suitable for deployment under field conditions to control the bacteria in the poultry sector [15,21,22].

In this sense, the use of bacteriophages (BP) has garnered increasing interest as a possible method to achieve *Salmonella* control in poultry farms in recent years, due to its high degree of specificity against the bacteria [22,23,24,25,26]. Among the different routes of BP therapy administration, the oral route is likely to be the most applicable in humans and animals [27,28,29]. The lack of BP stability upon exposure to acidic conditions (e.g., in the proventriculus/gizzard) and the relatively short residence times in the intestinal tract may limit the efficacy of orally delivered BP [30]. These limitations can however be overcome, such as through the co-administration of antacids [30,31] or through encapsulation of the BP [32,33,34]. Encapsulation provides protection from gastric acidity and could release high doses of the BP at the ceca, the predilected *Salmonella* site, incorporating approaches for burst release and/or sustained release; improving the efficacy of the orally administered BP [34,35,36]. Nevertheless, there is a need for better understanding of the stability and viability of the non-encapsulated and microencapsulated BP after being orally administered [37,38,39]. Therefore, the main objective of this study was to assess the release of the encapsulated BPs and the survival of *Salmonella* BP FGS011 (non-encapsulated and microencapsulated) through the chicken’s GIT under in vitro and in vivo conditions after oral administration to 1-day-old chicks.

## 2. Results

### 2.1. BP Characterization Assay

#### 2.1.1. BP Lytic Spectrum

The lytic host range against a panel of 13 strains indicated that BP FGS011 was able to lyse seven strains of *Salmonella* and one *Citrobacter* strain (Table 1).

#### 2.1.2. BP Thermal and pH Stability

**Figure 1:**
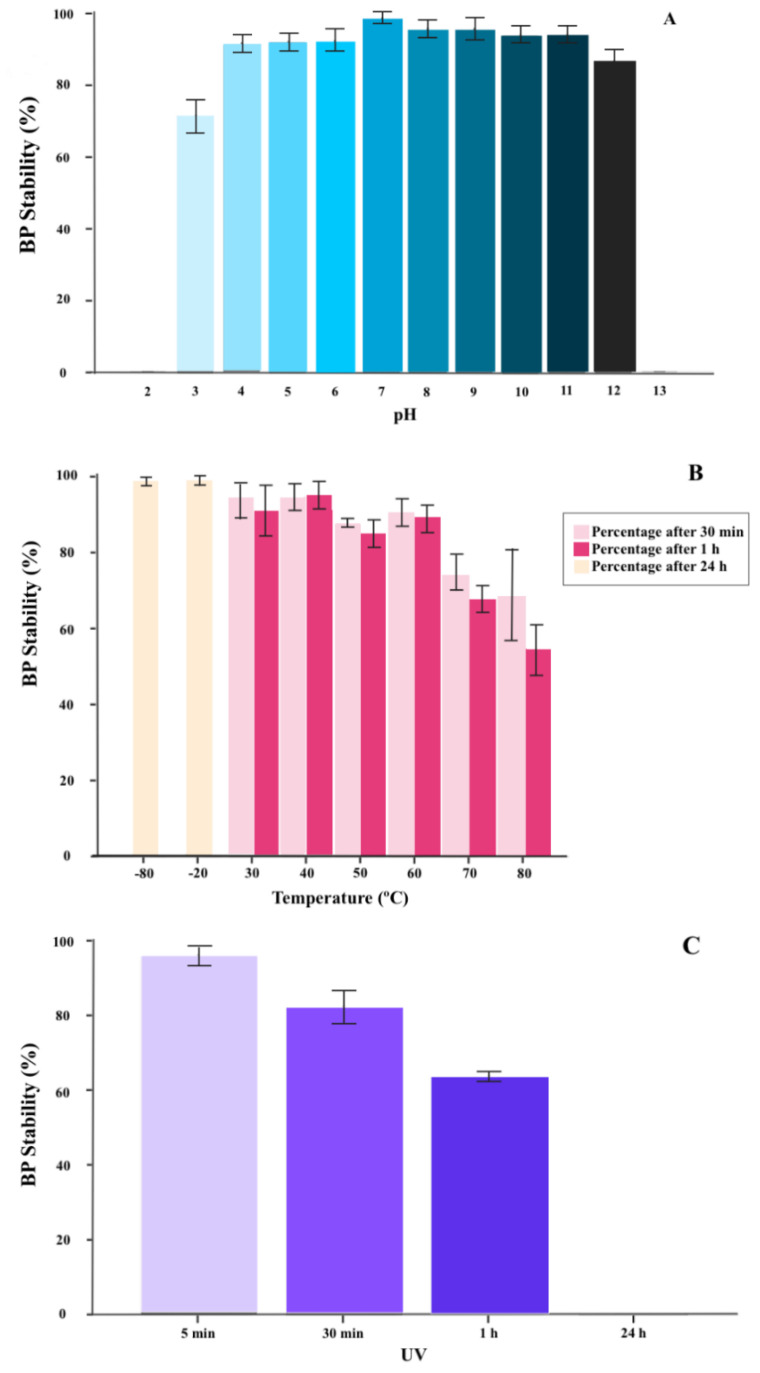
Stability of FGS011 exposed to different stress conditions. (**A**) pH stability of FGS011; (**B**) thermal stability of FGS011; and (**C**) stability upon exposure to UV radiation of FGS011. Data reported are means ± standard error of three independent trials. Error bars show standard error.

pH stability tests showed that FGS011 survived exposure to acidic pH as low as pH 4 and alkalinity as high as pH 11 after 2 h of incubation (Figure 1A). However, FGS011 titers declined when exposed to acidic pH less than pH 3 or alkaline pH greater than 12. The BP was rapidly inactivated at low pH 2 and high pH 13 (Figure 1A). Thermal stability tests indicated that BP FGS011 was relatively heat stable for up to 60 min at temperatures between 30 to 60 °C. However, the viable BP concentration decreased upon exposure to temperatures between 70 to 80 °C (Figure 1B). BP FGS011 also survived upon undergoing a freeze-thaw cycle at −80 °C.

#### 2.1.3. BP Inactivation with UV Radiation

The viability of BP FGS011 was maintained for up to 1 h of exposure to UV irradiation, nevertheless, no viable BP was measurable after 24 h of exposure (Figure 1C).

### 2.2. In Vitro Evaluation of the Release of BP under Different GIT Conditions (Experiment 1)

**Figure 2:**
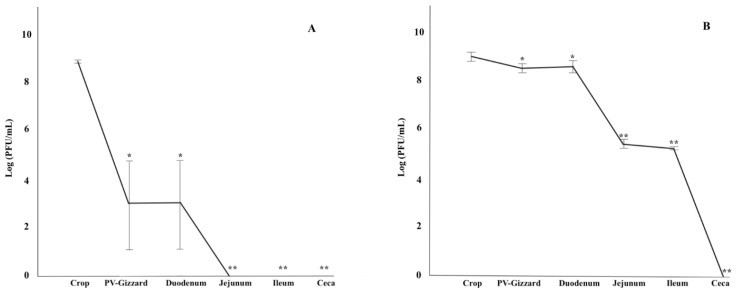
Amount of BP that remains encapsulated with the polymer Eudragit^®^ L100 (**A**) and Eudragit^®^ S100 (**B**) in vitro under simulated transit times and pH conditions of the crop (50 min at pH 5.5), proventriculus-gizzard (90 min at pH 2.5), duodenum (10 min at pH 5), jejunum (30 min at pH 6.5), ileum (70 min at pH 7), and ceca under anaerobic conditions (30 min at pH 8). Values are presented in Log10 (PFU/mL). L100: encapsulated BP with the polymer Eudragit^®^ L100; S100: encapsulated BP with the polymer Eudragit^®^ S100; PV: Proventriculus. Values shown are means ± standard deviations. Error bars show one standard deviation. The statistically significant differences in the count of BP that remain encapsulated throughout the simulated GIT with respect to the initial BP administered was represented as *, *p* < 0.001; and **, *p* = 0.0001.

Protection of encapsulation from GIT pH stress and BP release under different GIT pH conditions was evaluated for FGS011 encapsulated in formulations L100 and S100 simulating in vitro GIT conditions. All results represented in these experiments are with regard to the starting concentration of BP used in the experiment (10^10^ PFU). BP encapsulated in L100 was released in the crop due to the pH 5.5 (*p* < 0.05) (Figure 2A). However, BPs encapsulated in S100 did not release in the crop (pH 5.5), proventriculus-gizzard (pH 2.5), and the duodenum (pH 5) (Figure 2B). BP release was noted in the jejunum (pH 6.5), ileum (pH 7) and any residual encapsulated BP in the caeca (pH 8), indicating a statistically significant decrease along the lower GIT (*p* < 0.05) (Figure 2).

### 2.3. In Vitro Evaluation of BP Titres along the GIT (Experiment 2)

**Figure 3:**
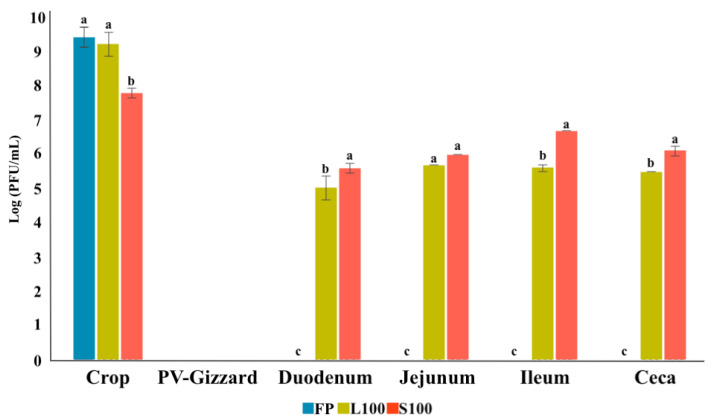
Amount of BP that remains in the supernatant whilst transiting across the simulated chicken GIT. Values are presented in Log10 (PFU/mL). FP: free BP; L100: encapsulated BP with the polymer Eudragit^®^ L100; S100: encapsulated BP with the polymer Eudragit^®^ S100; PV: Proventriculus. Error bars show one standard deviation. ^a,b,c^: different letters indicate statistically significant differences between groups in the count of BPs that released and survives throughout the simulated GIT at *p* < 0.05.

The survival of the BP (FP, L100 and S100) across the GIT was evaluated in vitro by passing the BP sequentially through each simulated GIT section (Figure 3). All results represented in these experiments are with regard to the starting concentration of BP used in the experiment (10^10^ PFU). The results showed that FP were inactivated upon exposure to proventriculus-gizzard conditions, and viable BP were not detected again in the following stages downstream of the proventriculus-gizzard. For L100, significant differences were observed along the simulated GIT sections (*p* < 0.05). BP released from L100 were detected in the crop, but no BP were detected in the neutralized supernatant of the simulated proventriculus-gizzard section. Thereafter, BP detection in the supernatant increased in the duodenum and BP titres were maintained in the jejunum through to the caeca (*p* > 0.05). For S100, the highest concentration of BP was detected in the crop (*p* < 0.05). No viable BP was detected in the proventriculus-gizzard. The concentration of BP in the supernatant increased in the duodenum and jejunum and was highest in the ileum with a 0.5 log decrease observed in the ceca (*p* < 0.05).

Moreover, statistically significant differences were observed among FP, L100, and S100 across the different controlled GIT conditions (pH, transit-time, and temperature) (Figure 3).

### 2.4. In Vivo Study of BP Survival along GIT (Experiment 3)

The distribution of FP, L100 and S100 within the GIT of 1-day-old chicks administered BP either in drinking water or in feed was determined after 24 h in the absence of *Salmonella* host. After 24 h, all the BP were present in all of the GIT sections (crop, proventriculus, gizzard, gut and ceca) of the treated groups. The control group was negative for the presence of BP in any of the samples analyzed.

From each free/released FGS011 (FP, L100 and S100), no statistically significant differences of BP concentration were observed through the different GIT sections (*p* > 0.05). For the different GIT sections, BP counts are summarized in Table 2.

On the other hand, statistically significant differences were shown between BP in the gut, with L100 being the BP to show the highest concentration (*p* < 0.05). However, no statistically significant differences were observed in the crop, proventriculus, gizzard, and ceca (*p* > 0.05) (Table 2).

For faecal samples, statistical differences were found in BP counts among the different groups (*p* < 0.05). The highest BP counts were in faeces of chicks fed L100 (6.4 Log10 PFU/g), followed by S100 (6.1 Log10 PFU/g) and finally FP (5.8 Log10 PFU/g) (*p* = 0.000).

## 3. Discussion

The survival of *Salmonella* BP FGS011 (non-encapsulated and microencapsulated) through the GIT under in vitro and in vivo conditions after oral administration to 1-day-old chicks was assessed. This is the first study to report the dynamics of the *Salmonella* BP encapsulated with the anionic polymers Eudragit^®^ L100 and Eudragit^®^ S100 through the chicken GIT.

Despite the NSCP, *Salmonella* continues to be a threat pathogen to the poultry sector and a potential risk from farm to fork [40]. Therefore, prevention and control are required throughout the poultry chain [12]. The negative economic impact of *Salmonella* for the poultry sector, the effects on human health, increasing antimicrobial resistance and the absence of effective alternatives for controlling *Salmonella* are drivers for exploration of the potential of BP therapy as a more effective biocontrol tool, reducing economic loses in the poultry industry and reducing the risk of food-borne diseases [15]. Oral BP therapy has been used as a tool for *Salmonella* control in the poultry industry [41,42,43]. Nevertheless, inherent challenges, such as the GIT environmental conditions, have led to divergent BP therapy results [32,37]. Although is this study both BP forms reached the ceca, previous studies reported a reduction in BP titres in vivo and suggested that this may be due to BP inactivation attributed to the acidic environment of the chicken’s gizzard. This may especially be the case for sensitive BP that do not survive low acidic pH exposure [32,37]. Previous studies have therefore highlighted the need for encapsulated BP that allow high titres of BP to be delivered to the cecum which is the most likely site of *Salmonella* colonization [37,44]. The encapsulation of BP and their controlled release may help in ensuring that the *in-situ* BP concentration remains at a therapeutically effective level, thereby allowing BP to amplify once the bacteria concentration increases to levels sufficient for in situ BP amplification [32]. The in vitro results of this study demonstrated that FP are likely to be inactivated upon exposure to proventriculus-gizzard pH (Figure 3). Whereas encapsulation of BPs significantly improved BP survival with S100 remaining encapsulated until the end of gut (Figure 2 and Figure 3). Encapsulation would therefore ensure that high BP concentration would be delivered to the caeca (Figure 3). Therapeutically effective levels reaching the intended *Salmonella* infection site, such as the lower intestine including caeca may improve BP therapy outcomes [32,38]. The current results indeed demonstrated that BP, both non-encapsulated and microencapsulated, appeared in the faeces, showing its high capacity of dissemination in the environment [22]. This matter will allow not only the control of the bacteria in the environment but also will limit the reinfection of the animals [22]. The reduction of faecal *Salmonella* shedding controlled by the NSCP, will entail an important economic reduction for the poultry sector [7,22].

Several complex external factors could influence the treatment success in vivo, such as the rapid clearance of the BP by active or passive host immunity, interactions of BP with the intestinal mucosa and with other gut microbiota [45,46,47]. The in vivo results obtained after BP administration in 1-day-old chicks demonstrated that encapsulated and non-encapsulated BP could survive through the GIT and were excreted in the faeces (Table 2). This fact may be explained due to the higher pH of the gizzard in the young animals (4.3 pH), which would allow not only the survival of the encapsulated BP, but also the survival of the FP (unpublished data) [48]. These results are in agreement with those obtained by Ma et al. (2016), who also observed that although FP did not survive gastric fluid conditions *in vitro*, in 1-day-old chicks BP were able to survive throughout the GIT, without no notable differences between free BP titres compared with the encapsulated form. Other authors have highlighted the possibility that for mature chicken GIT where the pH is lower, FP could not survive the gastric passage, and were not found to effectively reduce *Salmonella* numbers in the chicken intestine [49,50], compromising BP therapy outcomes in older animals [30,48].

Regarding the survival of BP formulated in different encapsulated forms administered to 1-day-old chicks, slightly higher concentrations of BP encapsulated in L100 were found to be delivered to the intestine compared with the S100 formulation (5.9 Log10 and 4.6 Log10 PFU, respectively). This may be due to lower amounts of BP released in the caeca of 1-day-old chicks from S100, which require higher pH levels to release BP [51,52]. BP therapy has previously been used to control *Salmonella* in poultry farms [22,23,24,25,26]. There is, however, a lack of available data at the field level [36]. Further studies are therefore needed to study the BP dynamics in the GIT throughout the six-week rearing period of broilers to assess the best way to incorporate phage in animal feed as well as the best intervention moment to prevent the spread of *Salmonella* in chicken farms.

## 4. Materials and Methods

All the animals were handled according to the principles of animal care published by Spanish Royal Decree 53/2013 [53].

### 4.1. BP Characterization Assay

BP FGS011 used in this study was isolated by Sevilla-Navarro et al. [40]. It was isolated from faeces samples from commercial poultry farms (broilers and layers) in Eastern Spain in the Poultry Quality and Animal Nutrition Centre of the Valencia Region (CECAV). The BP was stored at 4 °C until use. This BP was selected for this study due to its high lytic activity against its propagating strain and the wide lytic host range against *Salmonella* strains isolated from poultry farms. The bacterial strain used for BP isolation and amplification was *Salmonella* Senftenberg (*S.* Senftenberg), a field strain isolated from poultry farms during the NSCP [54] and selected from the collection repository of CECAV.

#### 4.1.1. BP Lytic Spectrum

In order to assess the lytic spectrum of the BP, the sensitivity of 13 field and reference bacterial strains including ten *Salmonella* serovars, one *Escherichia coli* strain, one *Citrobacter freundi* strain and one *Pseudomonas aeruginosa* strain were determined by spot test using the double agar method [40]. *Salmonella* serovars selected for BP lytic spectrum were those more prevalent in European poultry farms (*S.* Enteritidis, *S.* Typhimurium, monophasic *S.* Typhimurium, *S.* Infantis, *S.* Virchow, *S.* Kentucky, *S.* Ohio, *S.* Senftemberg, *S*. Indiana and *S.* Havana). Then, 200 µL of a log-phase culture of the bacterial suspensions in LB (Luria Bertani, VWR Chemicals, Barcelona, Spain) at an optical density (OD) 600 nm of 0.2 (~10^8^ CFU/mL) was added to 5 mL of LB agar (LB with 0.6% agar) tempered to 45 °C and poured onto previously prepared and dried LB basal agar (with 1.6% agar). The plates were dried in a laminar flow hood for 15 min. Subsequently, 10 µL of the BP FGS011 (10^10^ PFU/mL) suspension was spotted onto the surface of the double layer agar. The resulting plates were incubated overnight at 37 °C, and subsequently checked for the BP plaque formation on the bacterial lawns [40].

#### 4.1.2. BP Thermal and pH Stability

For thermal-stability testing, tubes with 100 μL of FGS011 (10^10^ PFU/mL) were mixed with 900 μL pre-heated sterile LB broth and were kept in a water bath ranging from 30 to 80 °C for 30 min and 60 min [41]. In addition, BP stability at −20 °C in a standard refrigerator, and at −80 °C in an ultra-low temperature freezer, was also evaluated for 24 h [55]. For pH stability testing, 100 μL of FGS011 (10^10^ PFU/mL) aliquots were mixed in a series of tubes containing 900 μL of sterile BPW (Buffered Peptone Water, VWR Chemicals, Barcelona, Spain) with varying pH values ranging from pH 2 to pH 13 (adjusted using NaOH or HCl) and incubated for 2 h at 37 °C [41,56]. After the incubation, ten-fold serial dilutions of the samples were carried out with LB and were plated by the spot test method and incubated 24 h at 37 °C. BP titration was performed per triplicate. The rates of BP pH/thermal stability were determined calculated with the formula: BP stability rate (%) = BP concentration (PFU/mL) under certain condition/initial BP concentration added (PFU/mL) × 100% [56]. These experiments were performed three times.

#### 4.1.3. BP Inactivation with UV Radiation

To quantify BP inactivation by UV radiation, 1 mL of FGS011 (10^10^ PFU/mL) was exposed to UV irradiation in glass petri dishes at room temperature for 24 h [57]. A 15-watt, low-pressure mercury germicidal lamp (U.V ESTERIL, J.P. SELECTA s.a.) with a sharp emission maximum at 230 nm was used as the UV source for irradiation of the BP. Samples were taken at different time intervals, at 5, 30, 60 min, and 24 h. Then, ten-fold serial dilutions of the samples were plated by the spot test method described above and incubated 24 h at 37 °C. BP titration was performed per triplicate. The rate of BP UV radiation inactivation was calculated with the stability rate (%) formula described above. This experiment was performed three times.

#### 4.1.4. BP Encapsulation

Encapsulation was performed according to Malik et al. [33]. For this study, two anionic polymers Eudragit^®^ L100, and Eudragit^®^ S100 were used for the BP encapsulation. Eudragit^®^ L100 dissolves at pH 6 and greater, while Eudragit^®^ Sl00 is less soluble than Eudragit^®^ Ll00 and dissolves at a pH of 7 or greater [58]. During this study, the BP FGS011 was evaluated without encapsulation as free BP (FP), and encapsulated with the polymers Eudragit^®^ L100 (L100) and Eudragit^®^ S100 (S100).

Eudragit L100 and S100 were kindly supplied by Evonik Germany. d-(+)-Trehalose dihydrate was purchased from Fisher Scientific (Loughborough, UK). Solutions containing different excipient (Eudragit S100, L100 with added trehalose) amounts were dissolved in 500 mL of deionised distilled water (dH_2_O). The ratio of S100 and L100 to trehalose was 2:1 and total solids content 12 g per 100 mL of solution.

In order to dissolve Eudragit, the pH of the water was changed to alkaline (pH 12) via addition of 4 M NaOH (Fisher Scientific, Hampshire, UK) to allow polymer dissolution, followed by pH adjustment to pH 7 using 0.1 M HCl prior to addition of trehalose powder, its dissolution, and further addition of bacteriophages to the solution. For each formulation, typically 10% (*v*/*v*) high-titre phage (~10^10^ PFU/mL) was added to the solution, yielding phage titres of ~10^9^ PFU/mL in the final formulations. The phage-containing solutions were spray-dried using a commercially available Labplant spray-dryer SD-06 (Labplant, UK Limited), which is a co-current dryer with a pneumatic atomiser and a cylindrical drying chamber of dimensions 215 mm outer diameter and 420 mm height. The air exit stream was passed through a high-efficiency particulate air (HEPA) filter prior to discharge. The diameter of the atomization nozzle used throughout the work was 0.5 mm with the measured feed liquid flow rate at 280 mL∙h^−1^ and a drying gas air flow rate of ~20 L∙s^−1^. The air inlet temperatures were set at 100 °C resulting in corresponding air outlet temperatures of 60 ± 2 °C respectively. The outlet temperature is only indicative of the highest temperature the phages could be exposed to as dry powders in the collection bottle. The temperature in the collection bottle varied between 40 and 60 °C.

### 4.2. In Vitro Evaluation of the Release of BP under Different GIT Conditions (Experiment 1)

**Figure 4:**
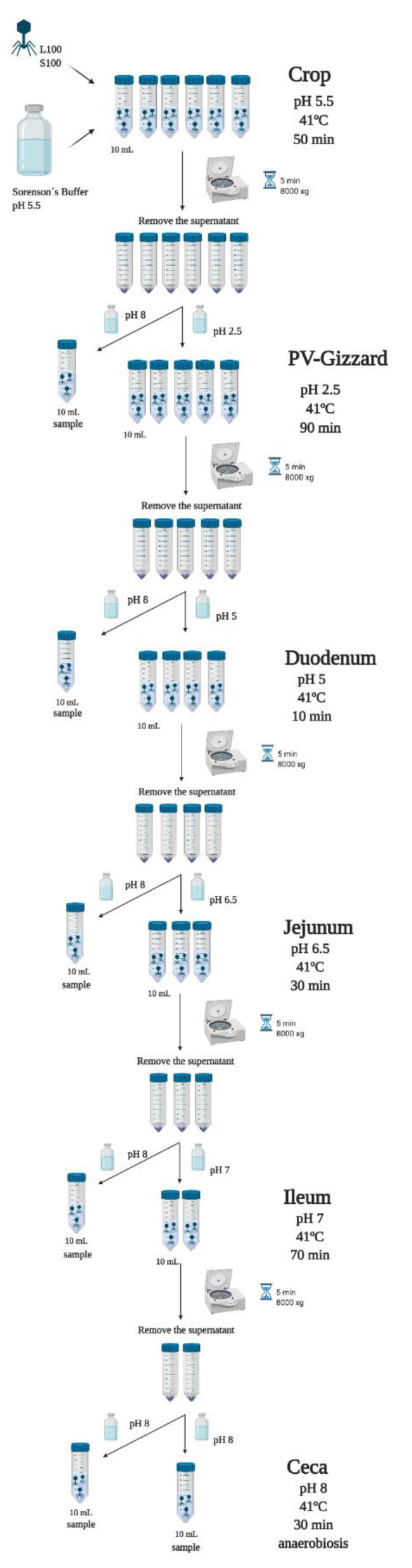
Scheme of the in vitro assay simulating the broiler GIT to assess the encapsulation maintenance (L100 and S100) along the GIT. The transit-time, temperature and pH of the crop (pH 5.5), proventriculus-gizzard (pH 2.5), duodenum (pH 5), ileum (pH 6.5), jejunum (pH 7), and ceca (pH 8 in anaerobiosis) were simulated. L100: encapsulated BP with the polymer Eudragit^®^ L100; S100: encapsulated BP with the polymer Eudragit^®^ S100. Created with BioRender.com.

To assess the release of the encapsulated BP (L100 and S100) along the GIT section, an in vitro assay was performed. For this, the amount of BP that remained encapsulated throughout the simulated GIT was analysed. The GIT conditions of the crop, proventriculus-gizzard, duodenum, ileum, jejunum, and ceca were simulated in vitro according to Ravindran [20] (Figure 4).

Firstly, to mimic the crop conditions, 10 mL aliquots of Sorenson’s buffer pH 5.5 was added to six falcon tubes for each of the encapsulated BP (L100 and S100). After that, an initial inoculum of 10^10^ PFU of each encapsulated BP (L100 and S100) was added into each respective tube. Samples were then incubated, with shaking, at 41 °C for 50 min. At the end of the incubation, all the tubes were centrifuged to sediment the phage containing capsules for 5 min at 8000× *g* and the supernatant was removed. In one tube for each BP, the capsule pellet was resuspended with 10 mL of Sorenson’s buffer at pH 8, and was vortexed and left for 10 min to allow the capsules to dissolve. After that, 100 µL of aliquot were taken for BP enumeration. From the remaining tubes, the pellets were resuspended in 10 mL of Sorenson’s buffer with adjusted to pH 2.5 with 1M HCl to mimic the proventriculus and gizzard conditions. The samples were incubated for another 90 min. After the incubation period, all tubes were centrifuged, and the procedure was repeated as described above. Hereafter, the process was repeated with 10 mL of Sorenson´s buffer at pH 5 for 10 min, followed by pH 6.5 for 30 min, and pH 7 for 70 min, to mimic the duodenum, ileum, and jejunum conditions, respectively. Finally, ceca conditions were mimicked using 10 mL of Sorenson´s buffer at pH 8 for 30 min under anaerobic conditions obtained with AnaeroGen Gas pack (Oxoid, Hampshire, UK, AN0035A) in anaerobic jars. Ten-fold serial dilutions of all the aliquots were carried out with LB and were plated by the spot test method and incubated for 24 h at 37 °C. BP titration was performed per triplicate.

### 4.3. In Vitro Evaluation of BP Titres along the GIT (Experiment 2)

**Figure 5:**
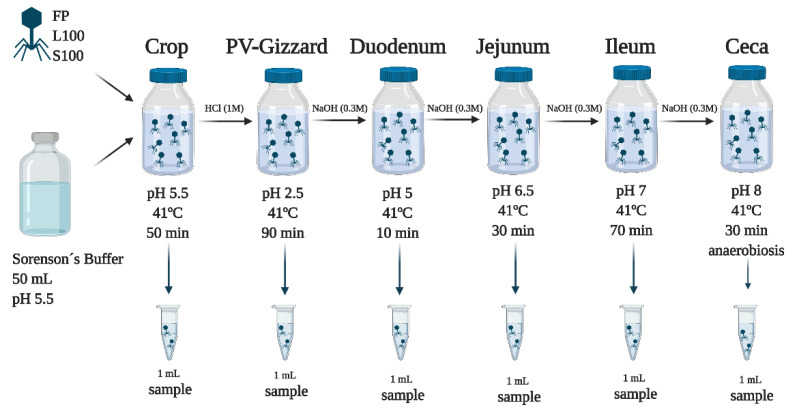
Scheme of the in vitro assay simulating the broiler to assess the release and survival of microencapsulated BP and the survival of non-encapsulated BP through the different pH, times and temperature conditions. The pH, transit-time and temperature of the crop (pH 5.5), proventriculus-gizzard (pH 2.5), duodenum (pH 5), ileum (pH 6.5), jejunum (pH 7), and ceca (pH 8 in anaerobiosis), were simulated. FP: free BP, L100: encapsulated BP with the polymer Eudragit^®^ L100; S100: encapsulated BP with the polymer Eudragit^®^ S100. Created with BioRender.com.

To assess the survival of the BP (FP, L100 and S100) across the GIT an in vitro assay was performed [37]. For this, the amount of released BP that survived throughout the simulated GIT was analysed. The BP (FP, L100 and S100) were passed sequentially through each simulated GIT section according to Ravindran [20]. To this end, the following stages were carried out (Figure 5).

Firstly, to mimic the crop conditions, 50 mL of Sorenson’s Buffer (pH 5.5) was inoculated with 10^10^ PFU of FP or 10^10^ PFU of encapsulated L100 and S100. Samples were then incubated, with shaking, at 41 °C for 50 min. At the end of the incubation period, 1 mL of aliquot was taken for BP enumeration. Then, to mimic the proventriculus and gizzard conditions, HCl (1 M) was added to each tube to adjust solution pH to pH 2.5 and the incubation continued for another 90 min. After this additional incubation period, 1 mL of aliquot was taken for BP enumeration and the procedure was repeated by adjusting the solution pH to pH 5 (using 0.3 M NaOH) for 10 min, followed by pH 6.5 for 30 min, and pH 7 for 70 min, to mimic the duodenum, ileum, and jejunum conditions, respectively. Finally, the ceca conditions were mimicked with pH 8 for 30 min under anaerobic conditions obtained with AnaeroGen Gas pack (Oxoid, Hampshire, UK, AN0035A) in anaerobic jars. At the end of each incubation, 1 mL of aliquot was taken for BP enumeration. The samples were centrifugated for 5 min at 8000 × *g* and the supernatant was taken. Ten-fold serial dilutions of the supernatants were carried out with LB and were plated by the spot test method and incubated 24 h at 37 °C. BP titration was performed per triplicate.

### 4.4. In Vivo Study of BP Survival along GIT (Experiment 3)

**Figure 6:**
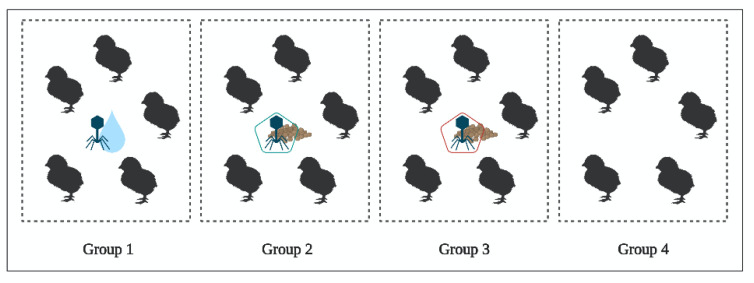
Experimental design of BP application in 1-day-old chicks. Group 1 received the FP via drinking water; group 2 received L100 via feed; group 3 received S100 via feed; and group 4 did not receive BP (control group). Created with BioRender.com.

An in vivo study of BP (FP, L100 and S100) survival along the GIT after oral administration of phages to 1-day-old chicks was carried out. Faecal shedding of BP was also measured. Twenty 1-day-old *Salmonella* free chicks (Ross 308) were randomly divided into four groups of five birds (Figure 6).

To assess the survival of the BP through the GIT, group 1 received 100 mL of 10^6^ PFU/mL of FP via drinking water, group 2 received 100 g of 10^6^ PFU/g of L100 via feed, group 3 received 100 g of 10^6^ PFU/g of S100 via feed, and group 4 did not receive any BP (control group). Then, 24 h after BP administration, animals from each experimental group (*n* = 5/group) were taken and the GIT was removed and processed for BP enumeration. The following sections were processed from each animal: crop, proventriculus, gizzard, gut, and ceca. Samples of each sections were collected under sterile conditions. The material used was sterile and it was changed between groups. Between samples, the material was sterilised with alcohol and fire [59].

Moreover, to assess fecal shedding, at least 10 g of faeces were taken from each experimental group. A total of 104 samples were weighed and homogenized in 1:10 mL of LB broth medium, centrifuged for 5 min at 16,000 × *g*, and the supernatant was filtered through a 0.45 µm membrane. Afterwards, 100 μL of each dilution was transferred to an empty well and ten-fold serial dilutions were performed using sterile dilution buffer (LB). Then, 10 μL of each dilution with 200 μL of the bacterial host suspension was mixed with 5 mL of LB 0.6% top agar layer and placed over a 1.6% LB agar bottom layer. Plates were incubated overnight at 37 °C. BP titration was performed per triplicate.

### 4.5. Statistical Analysis

Concentrations (PFU/mL) of BP were converted to Log10 (PFU/mL) and then averaged. A general linear model (GLM) was used to evaluate the release of encapsulated phage BP (L100 and S100) along the in vitro GIT conditions. GLM was used to evaluate the survival of the released BP (L100 and S100) and non-encapsulated BP along the in vitro GIT conditions. As a fixed effect, we included the gastrointestinal localization tract simulated (crop, proventriculus-gizzard, duodenum, ileum, jejunum and ceca), the initial BP concentration, and the BP (FP, L100 and S100). GLM was used to compare the activity of the BP (FP, L100 and S100) in vivo, including as a fixed effect, the gastrointestinal localization (crop, proventriculus, gizzard, gut, ceca, and faeces), and the BP (FP, L100, and S100). A *p*-value of *p* < 0.05 was considered to indicate a statistically significant difference. All statistical analyses were carried out using SPSS 16.0 software.

## 5. Conclusions

Significant differences were observed between phage delivery results of in vitro studies compared with in vivo results. In 1-day-old chicks there were no statistically significant differences between phage delivered along the GIT for the encapsulated and non-encapsulated phage (the gut being the exception, but differences were small here too). Encapsulation of the BP using the polymers Eudragit^®^ L100 and Eudragit^®^ S100 resulted in the delivery of phage in 1-day-old chicks with no adverse reactions observed in the animals. Further studies are needed to better understand the dynamics of the encapsulated phage released during transit through the GIT of the chickens during the entire production cycle.

## Figures and Tables

**Table 1:** The lytic spectrum of FGS011 against 13 bacterial strains from 4 genera.

Strain	Source/Reference	Lysis by BP FGS011
*Salmonella* Enteritidis	CCM160	+
*Salmonella* Typhimurium	CCM157	+
mST	CCM188	+
*Salmonella* Virchow	CECAV	-
*Salmonella* Ohio	CECAV	-
*Salmonella* Kentucky	CECAV	-
*Salmonella* Infantis	CECAV	+
*Salmonella* Senftemberg	CECAV	+
*Salmonella* Indiana	CECAV	+
*Salmonella* Havana	CECAV	+
*Escherichia coli*	CCM099	-
*Citrobacter freundi*	CCM091	+
*Pseudomonas aeruginosa*	CCM054	-

mST: monophasic *Salmonella* Typhimurium.

**Table 2:** In vivo comparation of BP treatments (FP, L100 and S100) within each GIT section.

	FP Log10 (PFU/g Content)	L100 Log10 (PFU/g Content)	S100 Log10 (PFU/g Content)
Crop	4.9 ± 0.4	6.3 ± 0.2	5.6 ± 0.3
Proventriculus	3.8 ± 0.4	5.7 ± 0.9	4.7 ± 0.7
Gizzard	4.5 ± 0.2	4.9 ± 0.5	4.6 ± 0.4
Gut	4.7 ± 0.2 ^b^	5.9 ± 0.5 ^a^	4.6 ± 0.5 ^b^
Ceca	5.2 ± 0.6	5.9 ± 0.2	4.7 ± 0.5

FP: free BP; L100: encapsulated BP with the polymer Eudragit^®^ L100; S100: encapsulated BP with the polymer Eudragit^®^ S100. Values shown are means ± standard error. ^a,b^ Different superscripts within each row indicates significant differences between means at *p* < 0.05.

## Data Availability

Not applicable.
